# Pro-Inflammatory Diet Is Correlated with High *Veillonella rogosae*, Gut Inflammation and Clinical Relapse of Inflammatory Bowel Disease

**DOI:** 10.3390/nu15194148

**Published:** 2023-09-26

**Authors:** Ilanna Marques Gomes da Rocha, Raquel Torrinhas, Danielle Fonseca, Clelia de Oliveira Lyra, Julianna Lys de Sousa Alves Neri, Bianca Depieri Balmant, Letícia Callado, Karen Charlton, Natalia Queiroz, Dan L. Waitzberg

**Affiliations:** 1 Laboratory of Nutrition and Metabolic Surgery (LIM-35), Department of Gastroenterology, Faculdade de Medicina, Hospital das Clinicas HCFMUSP, Universidade de São Paulo, São Paulo 05508-070, SP, Brazil; 2 Department of Nutrition, Universidade Federal do Rio Grande do Norte, Natal 59078-900, RN, Brazil; 3 School of Medical, Indigenous and Health Sciences, Faculty of Science, Medicine and Health, University of Wollongong, Wollongong, NSW 2522, Australia; 4 Department of Gastroenterology, Faculdade de Medicina, Hospital das Clinicas HCFMUSP, Universidade de São Paulo, São Paulo 05508-070, SP, Brazil

**Keywords:** dietary inflammatory index, ulcerative colitis, Crohn’s disease, gut microbiota, inflammation, calprotectin, zonulin

## Abstract

Inflammatory bowel diseases (IBD) are chronic conditions arising from an intricate interplay of genetics and environmental factors, and are associated with gut dysbiosis, inflammation, and gut permeability. In this study, we investigated whether the inflammatory potential of the diet is associated with the gut microbiota profile, inflammation, and permeability in forty patients with IBD in clinical remission. The dietary inflammatory index (DII) score was used to assess the inflammatory potential of the diet. The fecal microbiota profile was analyzed using 16SrRNA (V3–V4) gene sequencing, while fecal zonulin and calprotectin levels were measured with enzyme-linked immunosorbent assays. We found a positive correlation between the DII score and elevated calprotectin levels (Rho = 0.498; *p* = 0.001), but not with zonulin levels. Although α- and β-diversity did not significantly differ across DII quartiles, the most pro-inflammatory diet group exhibited a higher fecal abundance of *Veillonella rogosae* (*p* = 0.026). In addition, the abundance of some specific bacteria sequences showed an exponential behavior across DII quartiles and a correlation with calprotectin or zonulin levels (*p* ≤ 0.050). This included a positive correlation between sq702. *Veillonella rogosae* and fecal calprotectin levels (Rho = 0.419, *p* = 0.007). DII, calprotectin, and zonulin levels were identified as significant predictors of 6-month disease relapse (*p* ≤ 0.050). Our findings suggest a potential relationship of a pro-inflammatory diet intake with *Veillonella rogosae* and calprotectin levels in IBD patients in clinical remission, which may contribute to disease relapse.

## 1. Introduction

Inflammatory bowel diseases (IBDs) are highly prevalent chronic inflammatory conditions, including Crohn’s disease (CD) and ulcerative colitis (UC). Clinically, they are characterized by alternating sequences of active inflammation and remission. The etiology of IBDs appears to involves a complex interaction between host genetics, the intestinal microbiome, and immune factors, as well as environmental factors [1,2,3].

The clinical follow-up of IBD patients involves controlling disease activity. While endoscopy serves as the gold standard for evaluation, its impractical, expensive, and invasive nature limits routine use. As an alternative, calprotectin, a protein released by neutrophils, is a validated sensitive marker of subclinical mucosal inflammation [4], with elevated levels in feces associated with higher IBD activity [5,6,7]. Additionally, biochemical markers like C-reactive protein (CRP) have been used for systemic inflammation assessment [8], and zonulin has been considered as a potential marker of gut inflammation due to its role in regulating intestinal tight junctions [9].

The current treatment of IBDs often lacks effective outcomes with anti-inflammatory and immunosuppressive therapies [10,11]. Adjunct approaches, such as dietary interventions involving nutrients or foods with anti-inflammatory effects, have gained attention [12,13,14]. Diet is considered a potential modulator of the gut microbiome, which plays a crucial role in inflammation [15,16,17]. Some initial evidence suggests that interventions such as consuming fiber, prebiotics, and probiotics may benefit IBD patients [18,19,20].

Identifying the specific benefits of individual nutrients is challenging due to their consumption as part of a complete diet rather than in isolation. However, the Dietary Inflammatory Index (DII) may address this issue by assessing the overall inflammatory potential of a diet [21]. Derived from a comprehensive literature review of 1943 articles from eleven countries, the DII is scored based on prior knowledge of the pro-inflammatory and anti-inflammatory effects of 45 dietary components, allowing for international comparisons. Unlike other scores that assess adherence to established dietary guidelines or eating patterns, the DII stands out for its distinctive emphasis on inflammation [21].

Diet–microbiome interactions represent promising targets for modulating inflammation [15]. However, despite ongoing investigations into various dietary intervention strategies [19,22,23], the relationship between overall diet quality, gut bacteria, and markers of inflammation in IBD remains unclear. In this pilot study, we investigated whether the inflammatory potential of the diet is associated with the gut microbiota profile, inflammation, and gut permeability in patients with IBD experiencing clinical remission. Additionally, we assessed its potential in predicting disease relapse.

## 2. Materials and Methods

### 2.1. Ethical Issues

Our study protocol adhered to the ethical principles outlined in the Declaration of Helsinki, was registered at Plataforma Brazil (CAAE 01713018.0.0000.0068), and approved by the local Ethics Committee (CAPPesq 4.082.713). Prior to their inclusion, all the participants provided written informed consent.

### 2.2. Participants

Forty adult patients diagnosed with IBD were recruited from the Inflammatory Bowel Diseases outpatient clinic of the Department of Gastroenterology-Hospital das Clínicas, University of São Paulo School of Medicine (HC-FMUSP). The inclusion criteria were as follows: patients aged over 18 years, with a confirmed diagnosis of CD (*n* = 20) or UC (*n* = 20) and experiencing at least 1 year of clinical remission of their underlying IBD. CD and UC diagnoses were established in accordance with the European Crohn’s and Colitis Organization criteria [24,25], while data concerning disease remission were derived from medical appointments, which were conducted by the attending physician based on the Harvey–Bradshaw index (<5) for CD [26] and the partial Mayo score (<2) for UC [27]. We excluded participants who had a confirmed diagnosis of coeliac disease, irritable bowel syndrome, small intestinal bacterial overgrowth, previous gastrointestinal surgery, chronic inflammatory diseases, or infectious diseases. We also excluded those patients who had used antibiotics during the previous 3 months, were pregnant, had taken prebiotics or probiotics within the previous 2 months (except for consumption of dairy products and yogurt), used laxatives, followed special diets (e.g., vegan, intermittent fasting), or were receiving tube feeding. Finally, we excluded participants whose overall intake was below 800 kcal/day or above 4000 kcal/day for males, or below 500 kcal/day or above 3500 kcal/day for females.

### 2.3. Dietary Intake and DII Analysis

Data on food intake were collected through three rounds of a 24 h dietary recall (24h DR) covering three non-consecutive days, including one weekend day [28]. A trained nutritionist conducted the 24h DR to minimize any potential in-person variability. The dietary intake information was collected using home measures and converted into grams using Brazilian references [29]. The data were then entered into Easydiet, a professional software designed to determine the nutritional components of the diet. Energy, macronutrient, and micronutrient consumption were estimated based on the Brazilian Food Composition data (TACO). Additionally, the intake of flavones, flavan-3-ols, flavonols, flavonones, and anthocyanidins was estimated following the standards set by the US Department of Agriculture [30]. The multiple source method (MSM), a statistical modeling technique, was used to estimate the usual dietary intake of nutrients and foods in the 24h DR data and to adjust scores by energy [31,32]. Based on these data, we further calculated the DII for each participant by using the method developed by Shivappa et al. [21] to score 26 food parameters usually consumed by the Brazilian population: energy, 3 macronutrients (protein, carbohydrates, total fat, monounsaturated fat, and polyunsaturated fat), cholesterol, fiber, 13 micronutrients (folic acid, iron, magnesium, selenium, vitamins A, B1, B12, B3, B6, C, D, E and zinc), and 5 types of flavonoids (flavan-3-ol, flavones, flavonols, anthocyanidins, and flavonones). The daily intake of each parameter was divided by the global mean consumption’s standard deviation for standardization in a z score, which was further converted in a percentile, multiplied by 2, and subtracted from 1 to obtain a symmetrical distribution centered on zero, with limits of −1 (maximum anti-inflammatory) and +1 (maximum pro-inflammatory). The resulting value was multiplied by the inflammatory score of each specific component in the individual’s diet. We then calculated the individual global inflammatory index by summing the scores for all 28 parameters. Negative values indicated an anti-inflammatory diet, while positive values indicated a pro-inflammatory dietary pattern.

### 2.4. Demographic and Clinical Variables

At baseline, demographic and clinical data were collected for patients with IBD, including age, gender, physical activity, disease duration, weight, height, and measures of both fat and lean body mass. Anthropometric and body composition data were obtained during a morning examination while patients were in a fasting state. Body weight was measured using an electronic platform scale (Life Measurement Instruments, Concord, CA, USA), with patients wearing light clothing and no shoes. Body height was measured using a stadiometer (Sanny; American Medical do Brasil, São Paulo, Brazil), with patients standing barefoot, heels together, spine erect, and arms extended next to the body. Body mass index (BMI) was calculated as weight divided by height squared (kg/m^2^) and classified according to the World Health Organization (WHO) definition [33] as underweight (<18.5 kg/m^2^), normal weight (18.5–24.9 kg/m^2^), overweight (25–29.9 kg/m^2^), or obese (>30 kg/m^2^). Fat and lean body masses were measured using a bioelectric impedance device (QuadScan4000; Bodystat, Douglas, Isle of Man, British Isles), with the subject fasting, after urination and without jewelry or wristwatches beforehand. Patients with IBD were monitored for six months to evaluate routine clinical parameters and monitor for disease relapse.

### 2.5. Gut Microbiota Analysis

The α-diversity (Chao index) and β-diversity (Shannon index and Simpson index) of fecal bacteria, along with the species profile were analyzed. All the participants received a stool sample collection kit (Bioma4me, Sao Paulo, Brazil), which included detailed instructions to collect 1g of stool samples at home. Within 24 h of collection, the samples were transported to our laboratory using a specialized courier service under controlled temperature and stored at −80 °C until analysis. Total DNA was extracted from stool samples using the QIAamp^®^ PowerFecal^®^ DNA Kit (QUIAGEN^®^. Hilden, Germany), according to the manufacturer’s instructions. The 16S rRNA gene was amplified using V3 and V4-targed primers. The forward primer used for 16S Amplicon PCR was 5′TCGTCGGCAGCGTCAGATGTGTATAAGAGACAGCCTACGGGNGGCWGCAG. Amplicons were sequenced using the MiSeq platform (Illumina, San Diego, CA, USA). Subsequently, Raw sequence reads of the 16S rRNA gene were processed, trimmed, and assembled into amplicon sequence variants (ASVs). Each ASV represents a unique and exact sequence of a bacterium within its specific 16S gene (indicated as ‘sq.’). To assign species-level taxonomic IDs to each ASV, we employed DADA2, which identified exact matches (100% identity) between ASVs and reference sequences from the Silva database (version 132) [34,35]. The resulting ASV table allowed us to accurately identify and classify bacterial taxa.

### 2.6. Markers of Gut and Systemic Inflammation

#### 2.6.1. Fecal Zonulin and Calprotectin

The fecal zonulin concentrations were measured using the competitive ELISA method (Elabscience^®^ Biotechnology Inc., Baltimore, MD, USA), according to the manufacturer’s instructions. The spectrophotometric measurement was performed at 450 nm ± 2 nm to obtain the optical density (OD), which is directly proportional to the human zonulin concentration. The fecal zonulin concentration was then determined by comparing the OD of the stool samples to the OD of the standard curve. The obtained parameter was expressed in ng/mL with a sensitivity of 0.47 ng/mL, and the range of detection was 0.78–50 ng/mL. The fecal calprotectin concentration was measured using an enzyme-linked immunosorbent assay (ELISA; BÜHLMANN^®^ fCAL, Schönenbuch, Switzerland). For this assay, aliquots containing approximately 10 μL of centrifuged fecal extract, obtained from a 5 g stool sample within 24 h of collection, were used. The results were expressed as μg/g, with a measurement range of 20–8000 μg/g.

#### 2.6.2. Serum 25-Hydroxivitamin D [25(OH)D] and C-Reactive Protein 

Serum 25-hydroxivitamin D [25(OH)D] and C-Reactive Protein (CRP) concentrations were analyzed by chemo immune assay and immunoturbidimetry, respectively, according to the routine protocols for biochemical analysis of the HC-FMUSP Laboratory.

### 2.7. Statistical Analysis

Data normality was assessed using the Shapiro–Wilk test. Descriptive analysis determined the frequencies of categorical variables and presented continuous variables as mean ± standard deviation or median (minimum–maximum). Continuous variables between groups were compared using the Student t-test and Mann–Whitney U test, while the non-parametric Kruskal–Wallis test was applied to examine differences. The DII score was categorized into quartiles, ranging from the lower (Q1) to the higher inflammatory potential (Q4). Gut bacteria α-diversity was assessed using the observed ASV, Shannon diversity index, Chao1 index, and Simpson’s diversity index. Differences in α-diversity across DII quartiles were evaluated using the Kruskal–Wallis non-parametric test. The Mann–Whitney test with *p*-value adjustment according to the Benjamini–Hochberg procedure was used to identify significant differences between quartiles. Gut bacteria abundance was analyzed at the species level using the non-parametric Kruskal–Wallis and Mann–Whitney tests, and at the ASV level using expression analysis methods of genetic differential through negative binomial generalized linear models. Correlations between DII, altered gut bacteria, and markers of inflammation were tested using the Spearman correlation test. Furthermore, logistic regression analysis was performed to determine whether DII and other variables could predict 6-month disease relapse. The statistical analyses were conducted using the R program (version 2.5-7), with qualitative (diversity) and quantitative (abundance) analyses performed on gut microbiota species (Phyloseq package version 4.3) and gut microbiota ASV (DESeq2 package version 4.3). A significance level of 5% was adopted for all the statistical analyses. 

## 3. Results

### 3.1. Descriptive Data of IBD Patients

The cross-group descriptive data are available in Supplementary Table S1. Notably, more than half of the IBD patients (68.6%) exhibited low serum 25(OH)D levels (<30 ng/mL), while 27% presented high CRP concentrations (9/33). Increased calprotectin levels (>250 μg/g) were identified in 40.0% of participants. We found no significant differences in any demographic or clinical variables between UC and CD patients, including calprotectin levels. Furthermore, no significant differences were found for UC location or CD type and behavior. The mean DII score of IBD patients was 2.97 ± 0.89, ranging from −8.87 (maximum anti-inflammatory score) to +7.98 (maximum pro-inflammatory score). Supplementary Table S2 summarizes their dietary intake data across each DII quartile. Overall, nutrients that showed varying intake levels across the dietary inflammatory quartiles included fiber, flavanols, iron, magnesium, protein, vitamin C, and zinc.

### 3.2. Descriptive Data of IBD Patients According to Their DII Allocation

Table 1 presents the descriptive data of the IBD patients classified across each DII quartile. DII did not correlate with age, time of disease, height, weight, BMI, percentage of fat and lean mass, zonulin, vitamin D and CRP (*p* > 0.050). However, the higher the DII score the higher the fecal calprotectin concentrations in IBD patients, with patients in the Q4 (most inflammatory) having significantly more elevated concentrations of this biological marker of gut inflammation than patients in the Q1 (lesser inflammatory). In addition, DII exhibited a significant direct correlation with gut calprotectin concentrations (Rho = 0.498; *p* = 0.001).

### 3.3. GM Composition According to DII Allocation

**Figure 1:**
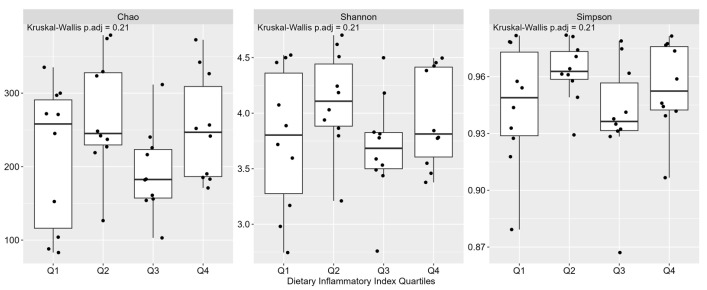
Gut bacteria diversity of patients (*n* = 40) with inflammatory bowel disease in clinical remission according to their allocation into dietary inflammatory index quartiles (Q).

Across the DII quartiles, IBD patients did not present significant differences in the α-diversity (Chao index) or β diversity (Shannon index and Simpson index) of fecal bacteria (Figure 1). On the other hand, in the comparative Phyloseq analysis for species level, *Prevotella stercorea* was significantly higher in Q2 (*p* = 0.002), while the *Veillonella rogosae* was significantly higher in Q4 (*p* = 0.026).

**Figure 2:**
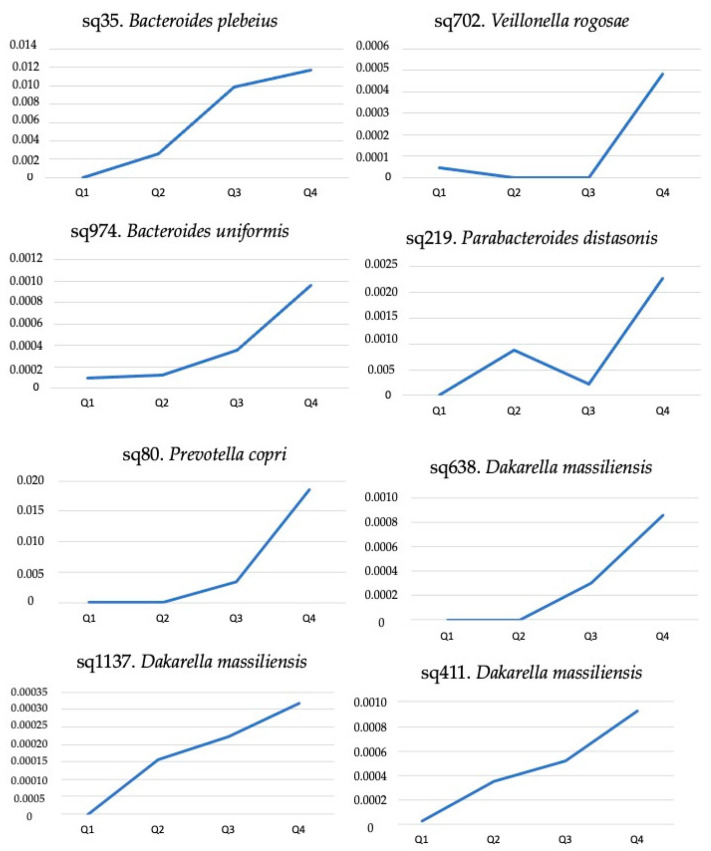
Exponential abundance of bacterial sequences that were present in 40 patients with inflammatory bowel disease in clinical remission according to their allocation into dietary inflammatory index quartiles (Q), which ranged from the lower (Q1) to the higher inflammatory potential (Q2).

The negative binomial generalized linear models performed (DESeq2) further showed an exponential behavior in the abundance of some bacterial sequences that were present in all patients across their DII quartiles (Figure 2). Among these, sq219. *Parabacteroides distasonis*, sq702. *Veillonella rogosae* and sq35. *Bacteroides plebeius* showed a positive correlation with calprotectin values, while three sequencies of *Dakarella massiliensis* (sq411., sq638., and sq1137.) showed a positive correlation with zonulin levels (Table 2).

### 3.4. DII, Gut Microbiota, and Inflammatory Markers in the Prediction of IBD Relapse 

Most patients were followed up to one year. At 6 months, 9 of 34 patients (26.5%) had progressed to active disease (relapse). Applying the logistic model (Table 3), high calprotectin, high zonulin, and a higher score of DII at baseline increased the chances of occurrence of 6-month disease relapse.

## 4. Discussion

Prior studies have established a link between IBD, dietary factors, and gut bacteria, indicating an intricate relationship between diet and the gut microbiota in influencing gut inflammation. [36,37,38]. Now, our prospective pilot study in IBD patients experiencing clinical remission has revealed a direct exponential relationship between gut calprotectin levels and the abundance of specific gut bacteria, based on the pro-inflammatory potential of their diet. Our findings suggest an intricate interaction among the inflammatory potential of diet, gut bacteria, and gut inflammation that may influence the progression of IBD.

Nutrients have the potential to distinctly modulate inflammation by affecting immunologic pathways, including cytokine secretion [39]. In our study, IBD patients in clinical remission were found to consume a pro-inflammatory diet (DII score 2.97 ± 0.89). Furthermore, participants consuming the most pro-inflammatory diet (Q4) exhibited higher calprotectin levels than patients with the least inflammatory diet (Q1), with DII showing a direct correlation with calprotectin concentrations. Our findings align with previous studies reporting a higher disease activity in IBD patients consuming a diet with higher inflammatory potential [40,41].

IBD patients have a lower-quality diet compared with healthy individuals, characterized by low intake of dairy, high-fiber foods, fruits, vegetables, and legumes [42]. This dietary pattern may be influenced by self-imposed restrictions due to concerns about exacerbating gastrointestinal symptoms or triggering disease relapse. However, we do not consume nutrients or whole foods in isolation but in combination; thus, the impact of diet on human health is likely to arise from the qualitative and quantitative fluctuations in daily meals. This may explain why some observations on the individual nutritional components consumed by our IBD patients did not align with conventional expectations, such as higher fiber consumption in the most inflammatory diet (Q4) compared with the lesser inflammatory diet (Q1). Upon closer examination, this finding was accompanied by a concomitant higher consumption of carbohydrates, which served as its primary source.

Therefore, our data highlight the significance of considering the entire dietary pattern assessed using DII, rather than individual nutrients, to determine the inflammatory potential of the diet. Accordingly, the mechanisms by which diets with high inflammatory potential affect IBD occurrence and development seem to be related to a combination of increased intake of pro-inflammatory dietary components and decreased ingestion of anti-inflammatory dietary components [43,44]. This imbalance may disrupt the gut microbiota, compromise the epithelial barrier function, and disturb intestinal immune homeostasis, ultimately triggering or exacerbating intestinal inflammation [45].

Interestingly, a recent study suggested a link between the inflammatory potential of the whole diet and the composition and function of the gut microbiome [15]. In our study, we found no differences in the α-diversity and β-diversity measures of microbial richness and evenness among patients assigned to different DII quartiles. Similarly, a previous study in patients with active IBD also failed to identify differences in the α-diversity of gut microbiota [46]. Our findings in IBD patients in clinical remission also included a significantly higher abundance of *Veillonella rogosae* in the patients with the most pro-inflammatory dietary intake, which was further linked to worsened gut inflammation, as measured by calprotectin.

*Veillonella rogosae* belongs to the *Veillonella* genus, which comprises several opportunistic pathogens commonly observed in oral and gut microbiota during bacterial infection-related diseases [47,48]. In IBD, the *Veillonella* genus is typically increased compared with healthy patients [49]. *Veillonella*’s lipopolysaccharide can stimulate pro-inflammatory cytokines, such as tumor necrosis factor-alpha and interleukin-6. Additionally, the abundance of this bacterial genus has been negatively correlated with lithocholic acid and deoxycholic acid, secondary bile acids known for their anti-inflammatory effects in the colon [48,50,51].

Dietary intervention in patients with Crohn’s disease is associated with maintenance in disease remission and is accompanied by changes in the fecal microbiome, including a decrease in *Veillonella* abundance [52]. Tian et al. [46] described a correlation between the *Veillonella* genus and the Crohn’s Disease Endoscopic Index of Severity and CRP values. The authors reported a higher abundance of *Veillonella parvula*, another species of the genus *Veillonella*, in IBD patients consuming a pro-inflammatory diet. Therefore, our study supports previous findings and highlights the potential role of *Veillonella*, specifically *Veillonella rogosae*, in increasing fecal calprotectin concentrations.

In addition to calprotectin, we also investigated the correlations of DII with other markers of gut (fecal zonulin) and systemic (CRP) inflammation. Nevertheless, no significant correlations were found between DII and these inflammatory markers. Instead, we observed certain sequences of other gut bacteria that exhibited exponential behavior across DII, which were correlated with calprotectin (*Parabacteroides distasonis*; *n* = 1) or zonulin (*Dakarella massiliensis*; *n* = 3) concentrations. 

Although *Parabacteroides* species are mostly commensals in the gut microbiota, some variants can display inflammatory potential and bacterial resistance [53]. To date, no previous reports have linked this specific variant to inflammatory markers. Regarding *Dakarella massiliensis*, which was isolated in 2017, there is limited research on its interaction with the human host, and no available information on inflammation and intestinal permeability. However, it belongs to the Sutterellaceae family and shares 92.4% genetic similarity with *Sutterella wadsworthensis* [54], a bacterium strongly associated with increased intestinal permeability [55,56].

While limited studies on serum and fecal zonulin concentrations as biomarkers of IBD are available and findings remain conflicting [8,57,58], CRP is currently used for IBD follow-up [8]. Our data on CRP levels contrast with the results of previous studies showing elevated concentrations in Crohn’s disease patients consuming a more pro-inflammatory diet (higher DII) [59]. However, as a systemic biomarker, serum CRP elevation may not be specific to intestinal inflammation and may lack sensitivity in detecting IBD patients experiencing clinical relapse of the disease [60,61].

To explore the association between a pro-inflammatory diet and clinical relapse, we applied a logistic regression model, revealing a significant effect of both DII and calprotectin in predicting a 6-month clinical IBD relapse. These findings align with previous studies reporting associations between a pro-inflammatory diet and increased disease activity in IBD patients [40,62,63].

A positive correlation between the DII score, gut microbiota, and disease activity was previously reported in patients with active IBD. Our study is the first to demonstrate that this association can also be observed in IBD patients during clinical remission and may contribute to disease relapse. Nonetheless, our study has certain limitations that should be highlighted. The relatively small sample size, particularly within subgroups, calls for caution when interpreting our findings. Moreover, the lack of endoscopic assessment and the use of a partial score to evaluate remission further underscore the need for confirmation through larger, well-designed cohort studies. 

## 5. Conclusions

In patients with inflammatory bowel disease in clinical remission, a pro-inflammatory dietary profile was associated with a high abundance of *Veillonella rogosae*, gut inflammation, and disease relapse, supporting the notion of a potential role of diet in shaping gut microbiota composition and local inflammation in this clinical population. 

## Figures and Tables

**Table 1:** Descriptive data of patients with inflammatory bowel disease in clinical remission according to their allocation into dietary inflammatory index quartiles.

Variable/Mean (SD)	Quartile 1 (*n* = 10)	Quartile 2 (*n* = 10)	Quartile 3 (*n* = 10)	Quartile 4 (*n* = 10)	*p* Value
DII	1.76 (0.45)	2.72 (0.24)	3.38 (0.21)	4.02 (0.32)	-
Age (year)	41.60 ± 14.20	46.80 ± 16.20	41.90 ± 14.70	49.00 ± 14.60	0.623
Time of disease (years)	11.00 ± 7.10	14.50 ± 5.00	9.70 ± 9.00	12.60 ± 10.20	0.309
Chao index	214.79 ± 97.46	270.58 ± 79.22	193.29 ± 57.89	252.08 ± 72.98	0.230
Shannon index	3.76 ± 0.64	4.11 ± 0.45	3.69 ± 0.46	3.95 ± 0.44	0.190
Simpson index	0.95 ± 0.03	0.96 ± 0.02	0.94 ± 0.03	0.95 ± 0.02	0.100
Calprotectin (μg/g)	127.90 ± 111.50	238.70 ± 245.10	406.80 ± 405.60	591.00 ± 338.10	0.013
Zonulin (ng/mL)	1.20 ± 0.10	1.20 ± 0.20	1.20 ± 0.20	1.30 ± 0.30	0.890
Weight (kg)	68.90 ± 15.90	72.50 ± 14.80	69.20 ± 9.70	71.1 ± 17.2	0.996
Height (m)	1.60 ± 0.10	1.70 ± 0.10	1.70 ± 0.10	1.70 ± 0.10	1.000
BMI (kg/m^2^)	25.50 ± 5.50	26.00 ± 3.30	25.10 ± 3.50	25.60 ± 4.40	0.949
Fat (%)	36.60 ± 9.20	32.00 ± 9.80	33.00 ± 8.30	31.10 ± 4.40	0.483
Lean mass (%)	63.40 ± 9.20	68.00 ± 9.80	67.00 ± 8.30	68.90 ± 4.40	0.483
Vitamin D (ng/mL)	28.00 ± 12.80	30.50 ± 9.80	25.40 ± 7.80	23.80 ± 8.50	0.448
CRP (mg/L)	3.20 ± 6.40	7.60 ± 11.40	3.30 ± 3.20	3.30 ± 4.40	0.629
Physically active *	11.00 ± 23.30	36.00 ± 64.50	65.00 ± 107.00	42.00 ± 93.00	0.830

Abbreviations: DII: Inflammatory diet index; BMI: Body mass index; CRP: C-reactive protein; * Minutes per week.

**Table 2:** Correlation between fecal zonulin and calprotectin concentrations and serum C-reactive protein levels with bacterial sequences showing exponential behavior towards the dietary inflammation index in patients with inflammatory bowel disease in clinical remission (*n* = 40).

Amplicon Sequence Variant	Zonulin		Calprotectin		C-Reactive Protein	
	rho	*p* Value	rho	*p*-Value	rho	*p* Value
sq80. *Prevotella copri*	−0.045	0.784	0.148	0.360	0.006	0.973
sq219. *Parabacteroides distasonis*	0.051	0.755	**0.343**	**0.030**	0.118	0.512
sq411. *Dakarella massiliensis*	**0.316**	**0.047**	0.142	0.383	−0.037	0.836
sq638. *Dakarella massiliensis*	**0.319**	**0.044**	0.281	0.078	−0.014	0.939
sq1137. *Dakarella massiliensis*	**0.486**	**0.001**	0.070	0.669	−0.206	0.250
sq702. *Veillonella rogosae*	0.039	0.811	**0.419**	**0.007**	−0.095	0.600
sq974. *Bacteroides uniformis*	0.068	0.677	0.113	0.486	0.098	0.588
sq35. *Bacteroides plebeius*	−0.076	0.641	−0.179	0.270	−0.087	0.629

Data were analyzed using the Spearman correlation test. Significant correlations are highlighted in bold.

**Table 3:** Multiple models for predictors of relapse disease activity in inflammatory bowel disease patients after 6 months (*n* = 34).

Variable	Adjusted Odds Ratio (95% Confidence Interval)	*p* Value
Calprotectin	1.000 (1.000–1.001)	0.00643
Diet inflammatory index	1.232 (1.046–1.451)	0.0177
Zonulin	2.300(1.102–4.800)	0.0336
CRP	1.019 (1.00–1.042)	0.1204

## Data Availability

Nucleotide sequence data used for this study are deposited in The European Nucleotide Archive (ENA) accession number PRJEB59338.

## Supplementary Materials

The following supporting information can be downloaded at: https://www.mdpi.com/article/10.3390/nu15194148/s1. Supplementary Table S1. Characteristics of patients with Inflammatory bowel disease (*n* = 40); Supplementary Table S2. Dietary intake data of IBD patients stratified in quartiles of the dietary inflammation (*n* = 40).
