# Supplementary material for: Pro-Inflammatory Diet Is Correlated with High Veillonella rogosae, Gut Inflammation and Clinical Relapse of Inflammatory Bowel Disease
Source: Nutrients. 2023 Sep 26;15(19):4148. doi: 10.3390/nu15194148 (PMC10574102; doi:10.3390/nu15194148)
Supplement: Supplementary file 1 [file nutrients-15-04148-s001.zip › nutrients-2579901-supplementary.pdf]

Pro-Inflammatory Diet Is Correlated with High *Veillonella rogosae*, Gut Inflammation and Clinical Relapse of Inflammatory Bowel Disease

Supplementary Table S1. Characteristics of patients with Inflammatory bowel disease (n=40).

Clinical Variable	CD (n = 20)	UC (n = 20)	p-value
**Age (years)**	43.15 (14.43)	46.50 (15.16)	0.607[1]
**Declared ethnicity**			
Caucasian	11 (55.00%)	12 (60.00%)	0.828[3]
Not-Caucasian	9 (45.00%)	8 (40.00%)	
**Gender (%)**			
Male	9 (45.00%)	7 (35.00%)	0.519[3]
Female	11 (55.00%)	13 (65.00%)	
**Weight (kg)**	69.80 (12.04)	71.08 (16.30)	1.000[1]
**Height (cm)**	1.66 (0.07)	1.65 (0,10)	0.665[1]
**BMI (kg/m²)**	25.17 (3.96)	25.93 (4.36)	0.735[1]
**BMI (kg/m²) (%)**			
Underweight	1 (5.00%)	0 (0.00%)	
Normal	9 (45.00%)	9 (45.00%)	0.699[2]
Overweight	6 (30.00%)	8 (40.00%)	
Obesity	4 (20.00%)	3 (15.00%)	
**Fat mass (%)**	32.95 (8.10)	33.34 (8.40)	0.829[1]
**Muscle mass (%)**	67.06 (8.10)	66.66 (8.40)	0.829[1]
**Bristol scale**			
1-2	1 (5.00%)	1 (5.00%)	0.924[3]
3 -4	12 (60.00%)	13 (65.00%)	
5-6	7 (35.00%)	6 (30,00%)	
**Evacuation frequency**	1,75 (0,78)	1.85 (1.34)	0.737[1]
**Smoking status (%)**			
No	20 (100.00%)	17 (85.00%)	0.231[3]
Yes	0 (0.00%)	3 (15.00%)	
**Alcohol use status (%)**			
No	13 (65/00%)	13 (65.00%)	1.000[2]
Yes	7 (35.00%)	7 (35.00%)	
**Disease duration (Years)**	11,3 (9.13)	12,6 (7.12)	0.379[1]
**Current medication use, n (%)**			0.073°
Aminosalicylates	3 (15.00%)	9 (45.00%)	
Immunosuppressors	5 (25.00%)	2 (10.00%)	
Biologicals	2 (10.00%)	5 (25.00%)	
Biologicals + Immunosuppressors	9 (55.00%)	3 (15.00%)	
No treatment	1 (5.00%)	1 (5.00%)	
**Zonulin (ng/mL)**	1.26 (0.21)	1.14 (0.15)	0.120[1]
**Calprotectin fecal(µg/g)**	424.8 (362.68)	257.4 (291.05)	0.123[1]
**C-reactive protein (mg/L)**	5.01 (8.69)	3.58 (4.12)	0.815[1]

Legend: ▪Mann-Whitney Test; #Fisher's exact test; *chi square test; IBD: Inflammatory bowel disease; UC: ulcerative colitis; CD: Crohn's disease.

Supplementary Table S2. Dietary intake data of IBD patients stratified in quartiles of the dietary inflammation (n=40).

Variable	Quartile 1 (n = 10)	Quartile 2 (n = 10)	Quartile 3 (n = 10)	Quartile 4 (n = 10)	p
Anthocyanidins (mg)	11 (18.6)	10.3 (11.6)	9.5 (5.2)	9.2 (3.7)	0.378
Carbohydrates (g)	206.3 (40.1)	249.6 (55.6)	225.6 (65.5)	249 (34.3)	0.074
Cholesterol (mg)	252.7 (92.6)	239.9 (67.2)	262.7 (71.9)	227.1 (41.2)	0.680
Energy (kcal)	1563.3 (326.5)	1859.7 (451.1)	1687.5 (475.7)	1844.7 (202)	0.278
Total fat (g)	57.7 (13)	58.7 (17.8)	58 (17.2)	63.3 (9)	0.839
Fiber (g)	14.1 (4.1)	22.1 (5.7)	17.3 (4)	28.9 (5.4)	0.000
Flavan 3-ol (mg)	6.3 (3.5)	7.7 (5)	13.3 (10)	14.2 (11.1)	0.071
Flavones (mg)	0.3 (0.2)	0.5 (0.4)	0.4 (0.3)	0.7 (0.8)	0.645
Flavanols (mg)	8.9 (4.8)	13.9 (6.3)	10 (5.7)	18 (7.8)	0.027
Flavanones (mg)	62.1 (88.3)	84.9 (110.5)	95.2 (106.9)	45.6 (74.4)	0.235
Folic Acid (µg)	85.6 (23)	84 (25.1)	104 (35.2)	76.6 (27.2)	0.354
Iron (mg)	6.6 (1.2)	9.5 (2.3)	8 (1.9)	9.2 (1.5)	0.005
Monounsaturated fat (g)	16.5 (3.2)	17.3 (4.5)	17 (4.8)	18.4 (2.6)	0.635
Magnesium (mg)	171 (31.8)	243 (39.8)	188.2 (39.7)	270.3 (31.6)	0.000
Polyunsaturated fat (g)	11.8 (4.7)	13 (4.8)	12.8 (5)	16.9 (4.4)	0.121
Protein (g)	57.6 (11.8)	79.1 (18.6)	68.9 (18.4)	74.8 (9.7)	0.013
Selenium (µg)	22.1 (6.1)	25.1 (5.1)	27 (8.2)	25.2 (10.6)	0.514
Vitamin A (RE)	282.2 (119.1)	344.9 (132.9)	297.6 (84.9)	472.4 (453)	0.831
Vitamin B1 (mg)	0.7 (0.1)	0.9 (0.2)	0.8 (0.2)	0.9 (0.2)	0.260
Vitamin B12 (µg)	1.6 (0.5)	1.7 (0.7)	1.7 (0.4)	2 (1.1)	0.949
Vitamin B3 (mg)	4.2 (1.5)	6 (2.6)	5 (1.5)	4 (3)	0.071
Vitamin B6 (mg)	0.7 (0.1)	0.7 (0.2)	0.7 (0.2)	0.8 (0.1)	0.579
Vitamin C (mg)	73.8 (24.9)	121.4 (48.2)	113.1 (54.1)	110.2 (24)	0.023
Vitamin D (µg)	1.9 (0.7)	2.3 (0.6)	2 (0.8)	1.6 (0.7)	0.312
Vitamin E (mg)	1.9 (0.4)	1.6 (0.5)	1.9 (0.8)	1.8 (0.6)	0.533
Zinc (mg)	6.7 (1.2)	9.4 (2.5)	7.7 (2.5)	9.8 (1.2)	0.003

RE, retinol equivalents.
